# Frailty in older adults admitted to hospital: a six-year dual-centre retrospective study of over 53 000 clinical frailty scale assessments

**DOI:** 10.1093/ageing/afaf137

**Published:** 2025-06-03

**Authors:** Emma Walker, Rebecca Winter, Luke Eliot Hodgson

**Affiliations:** Brighton and Sussex Medical School, 94 N - S Rd, Falmer, Brighton BN1 9PX, UK; Department of Medical Education Brighton, Brighton and Sussex Medical School, Brighton and Hove BN1 9PX, UK; Intensive Care Department, Worthing Hospital, Worthing, West Sussex, BN11 2DH UK

**Keywords:** frailty, clinical frailty scale, patients, older people

## Abstract

**Aim:**

To examine frailty assessments in older patients admitted to hospital, and explore associations with sex, admission and discharge status.

**Methodology:**

Worldwide, the prevalence of frailty is increasing. Stratifying frailty can be beneficial at a population level to improve public health and target local services. At an individual level, recognition of frailty can help inform prognosis and advanced planning. The Clinical Frailty Scale (CFS) is validated for predicting outcomes of older hospitalised adults.

All patients admitted into two hospitals in the South-East of England between 1 January 2017 and 31 December 2022, aged ≥65 years old with an electronically recorded CFS were included.

**Results:**

Over the study period there were 100 933 admissions, representing 53 361 individual patients. A single admission was observed in 16 284 (30.5%), whilst 37 077 (69.5%) had more than one admission. The mean CFS was 4.62 (SD 1.66) and 49.5% were living with frailty (CFS ≥5). Across 6 years, before, during and after the Covid-19 pandemic, this percentage remained stable. Females had a higher average CFS than males (4.74 vs 4.46, *P* < 0.01). Patients with a single admission had a higher mean CFS than patients with subsequent readmissions. Patients who died during admission had a higher average CFS than those who survived to discharge (6.02 vs 4.52, *P* < 0.01).

**Conclusion:**

This large cohort study of acutely admitted older adults found half were living with frailty. This highlights the importance of frailty identification to optimise personalised care. There was no significant change in frailty severity between 2018 and 2022.

## Key Points

- Worldwide, the prevalence of frailty is increasing, and stratifying this can be beneficial at a population level to improve public health and target local services.
- The Clinical Frailty Scale (CFS) is validated for predicting outcomes of older hospitalised adults. It uses a 9-point ordinal scale, with clinical descriptors and pictographs, and allows for reliable recognition of frailty state, based on clinical opinion.
- This study examined 100 933 admissions, representing 53 361 individual patients where the mean CFS was 4.62 and 49.5% were living with frailty (CFS ≥5).
- Patients with a single admission had a higher mean CFS than patients with subsequent readmissions and patients who died during admission had a higher average CFS than those who survived to discharge.

## Introduction

### Background

A factor associated with ageing and declining health is frailty, a syndrome encompassing accelerated deterioration in multiple physiological systems[1–7]. Higher levels of morbidity and disability drive attendances to healthcare facilities and subsequent hospital admissions [4, 8–10]. Stratifying frailty can be useful on both individual and societal levels [4, 8]. A frailty assessment can help identify vulnerability and inform plans for managing an individual’s predicted clinical course [4, 8, 10, 11]. Policymakers and local service providers may use information on the frailty status of the population to anticipate the range and extent of need, as previous studies have found that individual healthcare use increases with frailty [4, 8, 12, 13].

### Clinical frailty scale

The Clinical Frailty scale (CFS) is a well-validated tool for predicting the outcomes of older hospitalised people [8, 14–16]. It uses a 9-point ordinal scale, using clinical descriptors and pictographs, and allows for reliable recognition of frailty state, based on clinical opinion [8].

### Study aims

This study aimed to examine the CFS scores of patients during their first hospital admission within a 6-year period, including that affected by the Covid-19 pandemic (2017–2022). Differences observed between sexes, admission and discharge status were investigated.

## Methods

### Study setting

Data was collected from two district general hospitals in Sussex, South-East England, who provide acute care to a population of 450 000 residents [17]. The sites have ⁓950 inpatient beds [18].

### Data

#### Data collection

Patients were admitted to hospital after attendance to hospital, either through A&E or by GP referral. All ≥65s are expected to have a CFS recorded by a doctor as part of initial clerking for the purpose of an independent frailty assessment, found as part of the clerking proforma since 2016. This is deemed to be frailty status at 2 weeks prior to hospital attendance.

Doctors undertake training on completing the CFS on induction, and scores are checked and amended by consultant geriatricians within 24 h. The 2005 Version 1 of the CFS was used, where CFS 5 considers the individual to be living with frailty [8]. Patient-Track (Sydney, NSW, Australia) was used to record the screenings.

These assessments are routinely collected and electronically stored, which the Trust data analyst team retrospectively collated. All patient identifiable information was removed, and the set distributed securely to involved parties. Ethical approval was granted REC reference 18/SC/0513 IRAS 247109.

#### Sample

All patients ≥65 years at the time of attendance to A&E between the 1 January 2017 and the 31 December 2022 and who went on to have their first admission into hospital for at least one night.

### Statistical analysis

Statistical analysis was conducted using IBM SPSS statistics (version 29). Descriptive data is given as direct count with percentage or mean with standard deviation (SD). The Pearson X^2^ test was used to assess for difference between the distribution of two groups of categorical variables, and the Spearman ‘rho’ correlation coefficient for continuous variables. Where there were dichotomous variables, comparison of proportion between the two groups was conducted using the X^2^ test. Comparison of means between two groups of dichotomous variables was analysed using the independent sample T-test. Statistical significance was placed were *P* < 0.05.

## Results

Of the 100 933 admissions, 47 572 (47.1%) were excluded from primary analysis as they represented the readmissions for patients who had previously had an admission during the 6-year time frame (Appendix 1). 53 361 (52.9%) individuals were admitted into hospital.

The mean average CFS of all admission events was 4.62 (SD1.66) and mode 4. 49.5% of all patients included in the study were considered to be living with frailty (Table 1). The largest number of admissions was in 2017 (n = 11 845, 22.2%). The first year of the COVID pandemic, 2020, saw the lowest number of new patient admissions (n = 7532, 14.1%).

**Table 1:** Clinical frailty scale score for all patients by year, age, sex and admission status

Clinical Frailty Scale	1	2	3	4	5	6	7	8	9	*P*-value^a^	Mean average (SD)	*P*-value^b^
All data 100% (n = 53 361)	1.4 (741)	7.2 (3820)	21.0 (11 197)	21.0 (11 217)	16.5 (8797)	18.3 (9757)	11.6 (6165)	2.2 (1195)	0.9 (472)	**<0.01** ^**c**^	4.62 (1.66)	–
2017 22.2% (n = 11 845)	1.2 (146)	6.2 (731)	18.1 (2145)	20.3 (2400)	16.1 (1909)	19.8 (2340)	14.6 (1724)	2.7 (325)	1.1 (125)	**<0.01** ^**c**^	4.81 (1.69)	**<0.01** ^**d**^
2018 18.8% (n = 10 016)	1.8 (183)	7.7 (775)	21.1 (2113)	19.9 (1995)	16.7 (1669)	17.7 (1771)	11.9 (1187)	2.4 (242)	0.8 (81)	**<0.01** ^**c**^	4.59 (1.69)	**<0.01** ^**d**^
2019 15.5% (n = 8284)	1.6 (130)	8.0 (664)	21.4 (1776)	20.5 (1700)	16.3 (1354)	17.9 (1479)	11.1 (923)	2.4 (196)	0.7 (62)	**<0.01** ^**c**^	4.57 (1.67)	**<0.01** ^**d**^
2020 14.1% (n = 7532)	1.5 (116)	7.4 (557)	21.1 (1593)	21.8 (1641)	16.7 (1256)	18.1 (1363)	10.4 (781)	2.0 (147)	0.9 (73)	**<0.01** ^**c**^	4.56 (1.65)	**<0.01** ^**d**^
2021 15.1% (n = 8050)	0.9 (74)	6.9 (557)	22.8 (1835)	22.0 (1768)	16.8 (1353)	17.4 (1397)	10.4 (836)	2.0 (157)	0.9 (73)	**<0.01** ^**c**^	4.56 (1.61)	**<0.01** ^**d**^
2022 14.3% (n = 7634)	1.2 (92)	7.0 (536)	22.7 (1735)	22.4 (1713)	16.5 (1256)	18.4 (1407)	9.4 (714)	1.7 (128)	0.7 (53)	**<0.01** ^**c**^	4.51 (1.59)	**<0.01** ^**d**^
Age												
65–69 10.1% (n = 5409)	4.2 (226)	16.9 (913)	32.3 (1747)	20.4 (1105)	9.6 (517)	8.2 (441)	6.4 (348)	1.3 (68)	0.8 (44)	**<0.01** ^**c**^	3.76 (1.65)	**<0.01** ^**d**^
70–79 32.6% (n = 17 399)	2.0 (356)	11.1 (1926)	29.2 (5083)	22.5 (3908)	12.6 (2194)	12.2 (2131)	8.1 (1405)	1.4 (244)	0.9 (152)	**<0.01** ^**c**^	4.14 (1.64)	**<0.01** ^**d**^
80–89 38.8% (n = 20 722)	0.7 (144)	4.2 (873)	18.0 (3726)	22.6 (4680)	18.9 (3911)	20.3 (4199)	12.2 (2521)	2.4 (497)	0.8 (171)	**<0.01** ^**c**^	4.81 (1.56)	**<0.01** ^**d**^
90–99 17.8% (n = 9516)	0.2 (15)	1.1 (108)	6.7 (635)	15.8 (1500)	22.2 (2109)	30.4 (2891)	18.9 (1795)	3.8 (364)	1.0 (99)	**<0.01** ^**c**^	5.51 (1.37)	**<0.01** ^**d**^
100+ 0.6% (n = 315)	0.0 (0)	0.0 (0)	1.9 (6)	7.6 (24)	21.0 (66)	30.2 (95)	30.5 (96)	7.0 (22)	1.9 (6)	**<0.01** ^**c**^	6.08 (1.20)	**<0.01** ^**d**^
												
Female 54.9% (n = 29 316)	1.0 (290)	6.1 (1775)	18.9 (5551)	20.6 (6035)	17.8 (5211)	20.2 (5936)	12.3 (3612)	2.3 (684)	0.8 (222)	**<0.01** ^**e**^	4.74 (1.62)	**<0.01** ^**f**^
Male 45.1% (n = 24 045)	1.9 (451)	8.5 (2045)	23.5 (5646)	21.6 (5182)	14.9 (3586)	15.9 (3821)	10.6 (2553)	2.1 (511)	1.0 (250)	**<0.01** ^**e**^	4.46 (1.62)	**<0.01** ^**f**^
												
Single admission 30.5% (n = 16 284)	1.2 (200)	5.8 (946)	15.7 (2560)	17.1 (2778)	14.5 (2359)	20.2 (3290)	18.0 (2924)	5.1 (824)	2.5 (403)	**<0.01** ^**e**^	5.10 (1.81)	**<0.01** ^**f**^
Subsequent admissions 69.5% (n = 37 077)	1.5 (541)	7.8 (2874)	23.3 (8637)	22.8 (8439)	17.4 (6438)	17.4 (6467)	8.7 (3241)	1.0 (371)	0.2 (69)	**<0.01** ^**e**^	4.40 (1.54)	**<0.01** ^**f**^
												
Died during admission 6.2% (n = 3329)	1.2 (9)	0.8 (33)	1.8 (202)	3.9 (435)	5.5 (485)	7.7 (753)	13.5 (835)	31.4 (375)	42.8 (202)	**–**	4.52 (1.62)	**–**

^a^Indicating statistical comparison of distribution across CFS score.

^b^Indicating comparison of mean average between related comparable groups—such as between sexes or across decades of age. Significance is where *P* ≤ 0.05.

^c^Spearman ‘rho’ correlation coefficient.

^d^One-way ANOVA test.

^e^Pearson X² test. SD, Standard deviation.

^f^Independent samples T-test. Bold text indicating where a *p* value was significant.

The percentage of patients considered to have been living with frailty by each year included in the study (CFS ≥5) was 54.3% in 2017, 49.5% in 2018, 48.4% in 2019, 48.1% in 2020, 47.5% in 2021 and 46.7% in 2022.

The mean CFS differed between the years 2017 and 2022 but remained relatively stable. Compared using the One-Way ANOVA test, a statistically significant difference was found between the averages for each year (F(5,53355) = 45.41, *P* < 0.01). The effect size was found to be small (n^2^ = 0.004). However, further analysis was performed with Tukey’s HSD post-hoc. The mean CFS in 2017 was significantly higher compared to all other years, with *P* < 0.001. The mean average CFS score for 2018 was also significantly higher than the average for 2021 (*P* = 0.017, 95%CI 0.01–0.15). All other comparisons were statistically insignificant (*P* > 0.05).

### Analysis by sex

**Figure 1:**
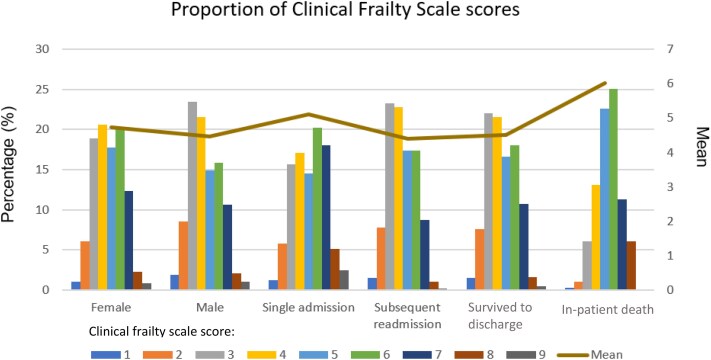
Showing the proportion of Clinical Frailty Scale scores by sex, admission status and discharge status.

A total of 29 316 females were admitted to hospital (54.9%), compared to 24,045 males (45.1%) (Figure 1). For females, the mean CFS was 4.74 (SD1.62). For males the mean CFS was 4.46 (SD1.62). 53.4% of females (n = 15 655) had a CFS ≥5 versus 44.5% of males (n = 10 721). Comparison of mean found a significantly higher average for females (*P* < 0.01, 95%CI –0.310 to −0.254).

### Analysis by admission status

A total of 16 284 (30.5%) patients had a single admission, and 37 077 (69.5%) patients had a subsequent readmission. For patients with one admission the mean average was 5.10 (SD1.81) and the mode 6. 60.3% had a CFS ≥5. For patients with a subsequent readmission the mean average was 4.40 (SD1.54) and the mode at 3. Only 44.7%, were considered to be living with frailty with a CFS ≥5. Comparison of means found the patients with a single admission had a significantly higher mean CFS score than those with subsequent readmissions (*P* < 0.01, 95%CI 0.671–0.731).

### Analysis by discharge status

Around 93.8% of patients (n = 50 032) were discharged from their hospital admission alive, compared to 6.2% (n = 3329) who died during admission. For patients discharged alive, the mean CFS was 4.52 (SD1.62). For patients who died during admission the mean was higher at 6.02 (SD1.64). Comparison of mean found the average CFS score for patients who died during their admission was significantly higher than for patients discharged alive (*P* < 0.01, 95%CI –1.550 to −1.436).

## Discussion

This observational study examined the CFS of older patients admitted into two UK district general hospitals over 6 years. This study investigated the largest number of events seen within comparable literature. The prevalence of frailty has been found to vary greatly between community and clinical situations throughout Europe [10, 15, 19–21]. 2017 estimates put the prevalence of frailty to be 38.9% [13], but this study found at least half of patients admitted to acute medical facilities are living with a degree of frailty. It is these patients who have a lower resilience and physiological reserve that are at greater risk of adverse events and significant deterioration in function [11, 22, 23]. Equally, as Walsh *et al*. found, healthcare utilisation, and subsequent expenditure, increases in direct correlation with severity of frailty [13].Identification of frailty allows for early intervention(s) to enhance the quality of care received, and to align it with person-centred treatment goals [11, 20, 23].

Across the 6-year timeframe, the only statistically different year was 2017, despite the dataset covering years directly affected by the Covid-19 pandemic. No other studies have examined the changes in CFS across multiple years in UK hospitals. The Covid-19 pandemic occurred within the timespan of this study and was found to have independently increased community frailty levels [23, 24, 25]. Importantly, this does not seem to have translated into the hospital environment, as the admission rate of those living with mild to severe frailty did not increase.

In this study, females were found to have a higher average CFS score, whilst a greater proportion of males were in the pre-frail category (CFS 3 or 4). This matches previous research findings, which highlighted that males spend more time in this category, and less living with frailty than females [26, 27].

The CFS is a validated tool for predicting readmission into hospital [12, 22, 23]. This study found the first recorded CFS score for patients who had subsequent readmissions into hospital were, on average, lower than for patients who only ever had a single admission. This could be explained by provision of interventions to prevent rehospitalisation for patients with more advanced frailty being implemented more appropriately or are more effective than for the cohort with lower CFS scores. It may be beneficial for further research to be carried out to identify individuals with risk factors for readmission, potentially further utilising the CFS. However, a CFS ≥6 has been found to be an independent predictor of inpatient mortality [26]. Therefore, patients with a single admission may have been more likely to die, thus confounding the data.

### Strength and limitations

This study represents the largest cohort of patients and admissions for the investigation of average CFS score. However, a limitation is the data is only from two hospital sites in the South-East of England. This does however form a baseline for further research to expand on and help begin a discussion on changes in frailty, both inside and outside the clinical environment.

Exploration of CFS score over time has not been undertaken before in the UK, particularly over the time of the Covid-19 pandemic. It would be beneficial to continue this examination to further map the impact this worldwide event had on the general health of the population.

This study did not consider primary diagnosis, nor admission under which clinical speciality, which could be considered a limitation as certain illnesses, especially those of acute of severe nature, may have a significant impact. However, the CFS was created to be an objective measurement of baseline frailty prior to admission, and therefore acute illness should not be relevant frailty status.

## Conclusion

This study of acute hospital admissions found half of all older patients were living with frailty. The distribution and average CFS score remained stable between 2018 and 2022, seemingly unaffected by the Covid-19 pandemic. Readmission rates were low in those with severe frailty. The mortality of admitted patients increased with higher frailty scores. Expansion of this project to further hospitals and healthcare environments, to aid generalisability would be beneficial.

## Supplementary Material

aa-24-2959-File002_afaf137
