# Supplementary material for: Frailty in older adults admitted to hospital: a six-year dual-centre retrospective study of over 53 000 clinical frailty scale assessments
Source: Age Ageing. 2025 Jun 3;54(6):afaf137. doi: 10.1093/ageing/afaf137 (PMC12131233; doi:10.1093/ageing/afaf137)
Supplement: aa-24-2959-File002_afaf137 [file aa-24-2959-file002_afaf137.docx]

**Frailty in older adults admitted to hospital: a six-year dual-centre retrospective study of over 53,000 Clinical Frailty Scale assessments**

# Appendix

Patient was discharged alive

**N = 12,955**

Patient was discharged dead

**N = 3,329**

≥65 admitted to hospital between 2017-2022

**N = 100,933**

Readmission events

**N = 47,572**

First admission

**N = 53,361**

Patients who only had a single admission between 2017-2022

**N = 16,284**

Patients who went on to have >1 admission between 2017-2022

**N = 37,077**

Key

Data excluded

Data included

*Figure 2 showing the number of patients admitted between 2017 and 2022, and the proportion of those who were readmitted or deceased.*

Number of patients by readmission status
